# Ceasing Intrathecal Therapy in Chronic Non-Cancer Pain: An Invitation to Shift from Biomedical Focus to Active Management

**DOI:** 10.1371/journal.pone.0049124

**Published:** 2012-11-08

**Authors:** Chris Hayes, Meredith S. Jordan, Fiona J. Hodson, Linda Ritchard

**Affiliations:** Hunter Integrated Pain Service, Newcastle, New South Wales, Australia; The James Cook University Hospital, United Kingdom

## Abstract

**Objective:**

To report long term experience (1997–2009) of intrathecal (IT) therapy for chronic non-cancer pain in the context of our team’s increasing emphasis on active management.

**Design:**

Descriptive case series.

**Setting:**

Australian tertiary multidisciplinary pain center, Hunter Integrated Pain Service (HIPS).

**Intervention:**

This case series reports the changing use of IT implanted drug delivery systems (IDDSs) for chronic non-cancer pain over 13 years. Initially IT therapy was used selectively following multidisciplinary assessment and double blind IT trial. Typical therapy combined opioid with clonidine. Multidimensional management was offered. Treatment strategy changed in 2003 due to HIPS experience of limited therapeutic gains and equivocal support for IT therapy in the literature. Subsequently IT therapy was no longer initiated for non-cancer pain and those on established regimes were encouraged to shift to oral/transdermal opioids with greater emphasis on active management. Patient education and consultation were key elements. Where IT cessation was elective gradual dose reduction commenced as an outpatient. In elective and urgent cases ketamine infusion and oral clonidine were used during hospital admissions to cover the switch to oral/transdermal opioids. Over the study period transition occurred to a broader management framework in which IT therapy for chronic non-cancer pain was no longer supported by HIPS.

**Results:**

25 patients were managed using IDDSs; 8 implanted by HIPS and 17 by other teams. Dose escalation and adverse effects were common. 24 of 25 patients ceased IT therapy; 7 (29%) with urgent IDDS related complications, 16 (67%) electively and 1 due to an unrelated death. The remaining patient returned to her original team to continue IT therapy. One post-explantation patient transferred to another team to recommence IT therapy. The remainder were successfully maintained on oral/transdermal opioids combined with active management.

## Introduction

Intrathecal (IT) drug delivery is a treatment option in the management of chronic non-cancer and cancer pain. It is also used for treatment of spasticity secondary to spinal cord or brain injury and disease. In chronic non-cancer pain and spasticity, implanted drug delivery systems (IDDSs) are generally used incorporating a catheter and pump. In cancer pain, cheaper IT portal systems are more commonly used in combination with an external pump.

IT therapy for cancer pain is supported by randomized controlled trials [1], [2] showing significant reduction in opioid toxicity and a trend toward decreased pain intensity and improved survival when compared to comprehensive medical management. IT baclofen therapy for spasticity has also shown benefit in randomized controlled trials [3], [4], [5]. The situation in chronic non-cancer pain is less clear. A review of the published literature in 1996 found 10 studies describing IT opioid therapy for persistent non-cancer pain [6]. The authors noted methodological limitations in all studies and concluded that the role of IT opioids in chronic non-cancer pain could not be determined from the existing literature. A systematic review in 2000 found 53 studies addressing both chronic non-cancer and cancer pain [7]. None were randomized controlled trials. Patients usually reported reduction in pain intensity and improvement in various quality of life indicators. The incidence of pharmacological side-effects was reported at 3–26% and mechanical complications at up to 20%. However, issues such as the use of variable drug regimes, heterogeneous patients, lack of comparative groups, and non-standard outcome measures were seen as problematic. The authors concluded that the “treatment is invasive, prone to side-effects and complications, costly and requires a large amount of technical support”. Furthermore they noted that they could not answer the question of whether or not IT opioid therapy was better than other existing treatments for chronic pain.

A more recent systematic review [8] of IDDSs for chronic non-cancer pain identified only 6 trials addressing both efficacy and complications. None of the 6 trials were randomized. Variable improvement in analgesia and function was reported. There was significant dose escalation and high risk of drug side effects and hardware problems. It was stated that “methodologic limitations precluded conclusions concerning the effectiveness of IDDSs in the long-term as compared with other treatments”. Another recent systematic review of IT therapy for non-cancer pain showed either Level II-3 or Level III (limited) evidence based on U.S. Preventive Services Task Force (USPSTF) criteria. Documented limitations of the study included paucity of literature, lack of quality evidence and lack of randomized trials [9].

Over time growing recognition of the extent of opioid side effects has led to questions about the safety of long term IT therapy. Suppression of the hypothalamic pituitary axis [10], [11], immunosuppression [12], [13], opioid induced hyperalgesia [14] and glial cell activation [15] are all of particular concern.

In addition to the multiple biological issues, there has been increasing consideration of the influence of psychosocial factors on outcomes of IT therapy [6]. This stems from the knowledge that psychological factors have a stronger influence on outcome than do biomedical factors in chronic pain generally [16].

In an attempt to implement a broader approach and improve outcomes, implanted devices (IDDSs or spinal cord stimulators) were combined with an intensive cognitive-behavioural pain management program [17]. Benefits in cognition, mood and disability were reported, as expected from participation in the pain management program alone, yet no change in pain intensity was noted. Given that traditional intensive pain management programs generally produce a modest reduction in pain intensity [18], [19], [20], [21] questions arise about the role of the implanted devices. One could argue that the implant cohort represented a more severely affected subgroup. On the other hand, there is the possibility that the use of the implanted devices may have interfered with the usual pain reduction achieved by the group pain management program. The authors [17] noted difficulties for participants with exposure to two potentially conflicting treatment models in terms of active or passive approach.

The development and application of a whole person model of care for persistent pain at Hunter Integrated Pain Service (HIPS) over the period 2003–2010 has been reported elsewhere [22]. The whole person approach emphasises active management and incorporates a fivefold focus on thoughts/emotions, actions, nutrition, personal story and biomedicine. The implementation of that approach had widespread effects across the service including a broadening of management for patients with IDDSs. Part of that changing focus involved a team decision in 2003 to phase out the use of IT therapy for chronic non-cancer pain due to our clinical experience of low efficacy and substantial risk of harm combined with limited scientific evidence supporting the therapy.

This study reports available outcome data for a small case series of chronic non-cancer pain patients treated with IDDSs in the context of our team’s evolving whole person model of care.

## Methods

### Ethics Statement

The project was discussed with John Hunter Hospital ethics committee and approval was given to undertake the work as an audit of clinical practice. The ethics committee supported our approach of not asking for patient consent to be part of the study. However, where patients underwent implantation of an intrathecal pump or related procedures, routine surgical consent was obtained. Patients also gave a general consent to provide serial outcomes measures which could to be used for research purposes. This became part of a standardised approach to outcome measurement used for all patients treated by HIPS in the latter years of the study.

### Participants

Participants included all 25 chronic non-cancer pain patients with IDDSs managed by HIPS in the 13 years from 1997 to 2009. Eight of these patients had their system implanted by HIPS while 17 were implanted by other specialist teams. Fifty six percent of patients were female, the average age at implant was 49 years (sd 13 years, range 20–73 years) and the majority had either private insurance (15 patients) or workers compensation cover (6 patients). The average duration of IT therapy in those who progressed to cessation was 6 years (range 2 months −13 yrs). The patient whose IT therapy lasted only two months had Type 2 diabetes mellitus and developed severe infection soon after implantation which necessitated IDDS removal.

In retrospect one can suggest that these patients may have received inadequate information about the potential harms of IT therapy and inadequate emphasis on the critical importance of simultaneously adopting active management strategies.

### Intrathecal Therapy

All patients implanted by HIPS attended multidisciplinary assessment and then had a positive response to double blind IT testing. This testing was done over 5 days via a temporary indwelling catheter. Patients were tested with higher and lower doses of morphine, clonidine and 2 saline placebos with each test substance running for 24 hours. Greater than 50% pain reduction was considered a positive response and these patients were then offered an IDDS as part of a comprehensive multidimensional approach incorporating active self management strategies. This rigorous assessment process was designed to maximise the likelihood of successful outcomes from the program despite the high risk of complications from IT therapy reported in the literature.

After implantation, the standard regime consisted of a continuous IT infusion (at times with programmed variability in infusion rate) of morphine or hydromorphone combined with clonidine. Bolus drug delivery was not used routinely.

For patients implanted at other centers variable selection and management approaches had been used prior to transfer to HIPS.

### Cessation of Intrathecal Therapy

From 2003 IT therapy was no longer initiated for chronic non-cancer pain and those on established IT regimes were encouraged to consider cessation and rotation to oral or transdermal opioids. This change process was supervised by a pain medicine specialist (CH) and co-ordinated by a nurse case manager. Physiotherapy and clinical psychology staff were available as required and 6 patients attended a high intensity (80 hours) cognitive behavioural pain management program. A patient centered approach was used with education and critical evaluation of the benefits and side effects of IT therapy. The combination of empathy with therapeutic boundaries was a key element of patient interactions and this was emphasised by the nurse attending each refill appointment.

From 2003–2007 patients were invited to consider IT cessation as part of an individualized case discussion process. In the final 2 years of the study a firmer management boundary was held and the few remaining patients with IDDSs were asked to choose between transferring to another specialist team willing to provide ongoing IT therapy or working with HIPS to achieve IT cessation. From 2003 there were alternative IT therapy providers available in the private sector in the same city and the public system in a neighbouring city 2 hours drive away.

Once a patient made the decision to move towards cessation of IT therapy, gradual dose reduction (eg. 5–10% reduction in daily dose at each refill) on an outpatient basis was commenced. A planned inpatient admission was then arranged for the final tapering and cessation of the greater part of the IT dose. This was done in a daily stepwise fashion under cover of subcutaneous or intravenous ketamine infusion (up to 20 milligrams per hour) with adjuvant oral clonidine (100–150 micrograms 8^th^ hourly). A daily reduction of approximately 20% of the admission IT dose was typical in the inpatient setting. As the IT therapy was tapered an alternative oral or transdermal opioid was initiated and built up to target dose. If there was evidence of opioid withdrawal this was managed by increasing the dose of ketamine or oral clonidine, slowing the rate of reduction in IT opioid or increasing the dose of oral or transdermal opioid. Duration of the inpatient admission was usually one week. Following elective cessation of IT therapy, patients were encouraged to consider explantation of the non-functioning IDDS after approximately 6 months.

In some situations cessation of IT therapy was abrupt due to development of urgent complications. In these cases inpatient ketamine infusion and oral clonidine were also used to cover initiation of an oral or transdermal opioid regime.

## Results

From the cohort of 25 patients managed with IDDSs, 24 ceased IT therapy. The remaining patient returned to her team of origin to allow continued IDDS usage. One of the 24 patients in whom IT therapy was ceased (in this case due to an urgent infective complication requiring IDDS removal) elected after 12 months to transfer to another specialist team in order to access a new IDDS.

Sixteen (67%) of the 24 patients who stopped IT therapy did so electively after a process of education and consent. In some instances, this preparation took up to 18 months. In one case tapering and cessation of IT dose was medically mandated as a result of aberrant opioid use related to factitious disorder (described in greater detail below). In 7 cases (29%) IT therapy was ceased abruptly due to urgent complications related to the IDDS. Complications included a pump malfunction due to gear failure combined with catheter occlusion, a septum failure (resulting in life threatening overdose), ulceration of the IDDS through the abdominal pocket, an IT granuloma associated with spinal cord compression and 3 severe infections requiring pump removal (patient interference relating to previously undiagnosed factitious disorder contributed in 1 of these cases). Despite the severity of these complications only 1 of the 7 patients reported long term sequelae; the person with the IT granuloma developed marked leg weakness and urinary dysfunction and became wheelchair dependent. In 1 elderly patient IT therapy was ceased due to death from a pre-existing cardiac condition.

By the end of the study period in December 2009, 20 of the patients in whom IT therapy was ceased had progressed to explantation of the IDDS. Usually this occurred within 6–12 months of ceasing IT therapy although one patient waited 3 years before deciding to go ahead with explantation. Two patients still had the IDDS in situ but inactive. Both were considering explantation.

Opioid doses were monitored at key time points over the course of therapy. Daily oral morphine equivalent dose was calculated for each patient pre-implantation, immediately after cessation of IT therapy and at later review (3–12 months). Dose conversion ratios used are listed in the “Opioid use in persistent pain” guideline on HIPS website [23]. Daily IT morphine equivalent dose was calculated for each patient after initial stabilisation on IT therapy and then just prior to reduction and cessation of the regime. IT hydromorphone dose was multiplied by a factor of 5 to convert to IT morphine equivalent. Due to uncertainty in the literature in regard to equivalence ratios of IT to oral morphine this conversion was not undertaken for data presented.

Due to the small sample size, the non-parametric Wilcoxon Signed Rank Test was used to compare opioid dose at different times. There was a statistically significant increase in average daily IT morphine equivalent dose over the course of IT therapy [Z(15) −3.195, p = 0.001, mean difference 5.76, sd 7.365] from 1.83 mg (sd 12.56) to 8.78 mg (sd 8.45). There was no significant change in average daily oral morphine equivalent dose before or after IT therapy [Z(14) −0.659, p = 0.510, mean difference 30 (sd 150)], from 143 mg (sd 136) to 133 mg (sd 99). A maintenance average daily oral morphine equivalent dose was also calculated at later review (3–12 months post cessation of IT therapy). This reduced slightly to 123 mg (sd 133).

In regard to patient health care utilization at HIPS, after exclusion of one extreme outlier, non parametric statistics (the Wilcoxon Signed Rank Test) were used to ascertain whether there was a change in the number of appointments attended following IT therapy cessation. There was a statistically significant decrease in the number of appointments at HIPS in the year following IT cessation (4.00 appointments, sd 3.07) compared with the year before IT cessation [12.35 appointments, sd 8.45: mean difference = 8.19, sd 7.27, Z(15) −3.05, p = 0.002].

Fifteen of 24 patients who ceased IT therapy completed the standard HIPS outcome questionnaire after cessation. Outcome measures addressed pain severity, pain interference, psychological distress, self efficacy, health care utilization, employment status and satisfaction with care (Table 1). Time frame was not standardised, with data collected on average 23 months after cessation (range 1 month to 6 years). On average, patients rated pain severity and interference in the moderate range (Brief Pain Inventory) [24], psychological distress in the normal range (Kessler10) [25], self efficacy in the moderate range (Pain Self Efficacy Questionnaire) [26], and 33% were employed. Health care utilization due to pain averaged 8 visits to health professionals of any sort in the 3 months prior to questionnaire completion. Seventy seven percent of patients were either satisfied or very satisfied with their care. Unfortunately insufficient data was available to compare the above results with standard measures prior to the cessation of IT therapy (N = 5) or prior to IDDS implantation.

**Table 1:** Standard measures data post cessation of IT therapy.

Measure	Mean (sd)	Patients (n)
Pain Severity (BPI)	5.2 (1.87)	15
Pain Interference (BPI)	4.91 (2.34)	12
Self Efficacy (PSEQ)	32 (11)	13
Psychological Distress (K10)	19 (10)	14
Health Care use (attendances in past 3 months)	8 (7.75)	16
Work Status	20% Full time	15
	13% part time	
	20% home duties	
	47% retired	
Patient satisfaction (1–5)	8% very unsatisfied	13
1 = very unsatisfied	15% neither satisfied or unsatisfied	
5 = very satisfied	15% satisfied	
	62% very satisfied	
Current side effects (0–10)	43% no side effects	14
0/10 = no side effects	43% low to moderate side effects (1–3/10)	
10/10 = severe side effects	14% high side effects (8 and 9/10)	

In order to gain insight into the patients’ perspective of ceasing IT therapy, qualitative data was gathered retrospectively from 17 of 24 patients via a brief questionnaire (postal return or telephone discussion). Table 2 details the difference in patient predicted and actual pain intensity in the 3 months following IT therapy cessation. It can be seen that more patients predicted the outcome of IT therapy cessation to be worse than it actually was in regard to pain intensity. Of the 5 patients reporting pain intensity as “much worse” following cessation of IT therapy, 1 had factitious disorder, 1 had the IDDS for only 2 months before requiring urgent removal because of infection and 3 had difficulty transitioning away from a biomedical focus and required ongoing supportive case management.

**Table 2:** Predicted versus actual pain intensity 3 months after ceasing IT therapy.

Pain intensity after IT cessation	Predicted (Number of Patients)	Actual (Number of Patients)
“Lots better”	2	1
“A bit better”	1	1
“No difference”	1	4
“A bit worse”	8	6
“A lot worse”	5	5

Disadvantages reported by patients related to cessation of IT therapy included: transient withdrawal symptoms, increased pain, constipation from oral opioids and reduced physical activity. However, the HIPS team noted some inconsistencies in patient reporting of activity. For example, at times patients stated that reduction in activity followed IT cessation when in fact the problem was longer standing.

Advantages reported by patients following IT therapy cessation included: reduced side effects such as ankle oedema, sweating and weight gain (some patients lost 20–40 kg after stopping IT therapy); no longer needing testosterone replacement; return of menses in pre -menopausal women; unassisted conception; loss of discomfort from the mass effect of the IDDS; loss of worry about potential complications, technical problems and future costs of refills and IDDS replacement; fewer visits to a pain management center and less time off work for refills and associated treatment.

Two patients were diagnosed with factitious disorder after implantation. One of these was implanted by the HIPS team and one transferred from another service. Both had IT therapy ceased after the development of aberrant behaviors. As noted above, one of these patients consistently interfered with the IDDS which produced infection around the pump and along the catheter track. The other patient falsely reported a cancer to his GP who made a subsequent referral to the local Palliative Care service. The patient did not mention his IDDS and a subcutaneous opioid infusion was commenced. This in combination with the unrecognized IT opioid regime produced respiratory depression and aspiration requiring urgent hospital admission and opioid antagonist treatment.

## Discussion

HIPS approach to IT therapy for chronic non-cancer pain changed over the 13 years of the study in the context of an evolving whole person approach that incorporated a greater emphasis on active management [22]. There was a transition from selective use of IDDSs at the beginning of the study period to avoidance of that form of therapy by the end. There was a parallel transition in case management style as the HIPS team steadily gained confidence in promoting the message that good quality of life was possible without an IDDS. What started as a more tentative exploration of the advantages and disadvantages of IT therapy ended in a much more confident prognostication of the feasibility of cessation. Our clinical impression of IDDS use was of modest analgesic benefit in the initial 6 months of therapy which declined over the longer term. In addition, while IT therapy continued we observed a consistent lack of functional improvement, a pattern of passivity in regard to self management and significant reinforcement of the illness role.

In the overall analysis of IT therapy consideration must be given to the high risk of serious complications (29%) and the multiple less serious side effects demonstrated in this cohort of patients. Such findings reflect those described elsewhere in the literature [7]–[11] and raise questions about the acceptable balance of risk and benefit in managing a non-life threatening condition in a vulnerable and complex patient group.

Appropriate case selection is commonly raised as a key issue in the literature. The importance of psychological screening and assessment of patients for IT therapy has been emphasised [27]. However the assessment process can be difficult even with skilled staff as illustrated by the diagnosis of 2 cases of factitious disorder post implantation in our cohort. In both cases this was missed at initial multidisciplinary assessment pointing to the challenge of making the diagnosis at a typical one off psychological assessment without the benefit of observation over time. In addition to clinical assessment challenges there are financial issues related to the high cost of IT therapy. In our cohort 84% of patients were covered by private insurance or workers compensation. At a practical level this may simply reflect the poor access of publicly funded patients to IDDSs. However, at another level there is the possibility that the availability of private and workers compensation funding might have influenced team decision making in favour of more expensive, but not necessarily more effective treatment strategies.

Escalation of IT dose was clearly demonstrated in this patient cohort. However it was interesting to note that following cessation of IT therapy, doses of oral/transdermal opioids returned to pre-implant levels or lower. Comparison of IT with oral/transdermal doses across the study period is hampered by uncertainty about dose equivalence with reported conversion ratios of IT to oral morphine ranging from 300∶1 [28] to 12∶1 [29].

The ideal drug combination for IT therapy is not known. In our cohort the development of tolerance appeared to be a major limiting factor. Opioid induced hyperalgesia may also have contributed. In part these problems may have related to the regular IT opioid usage of only morphine or hydromorphone as compared to the broader range of rotational options available for oral/transdermal opioid delivery. In the future it is possible that the use of additional IT agents such as ziconotide, local anaesthetics and alternative opioids may improve outcomes as suggested by the consensus guidelines of an expert panel [30], but there is currently a lack of evidence to support such biomedical therapeutic optimism. There also remains the unanswered question of whether any specific patient sub-groups can be identified that are more responsive to IT therapy.

As described above, our team’s clinical impression was that patient passivity in regard to their own management was widespread despite a major effort to promote IT therapy as simply one part of a multidimensional approach. A notable trend was captured during data analysis that illustrated this problem. When linked psychology or physiotherapy appointments were made on the same day as the IDDS refill, the allied health appointments were often cancelled by patients even though they still attended the refill. The lure of “advanced” technology proved seductive and distracted patients from active self-management. Patients readily became psychologically dependent on the IDDS. In every case there was a difficult phase of coming to terms with the idea that life without the device was possible. This proved to be the most critical issue in the process of cessation of IT therapy. Once the patient came to a point of acceptance of the possibility of cessation, the practicalities at both organisational and pharmacological levels were generally straightforward. This included an increased capacity to engage with active management as judged subjectively by our team.

While commencement of IT therapy did not appear to significantly improve patient functioning, cessation of IT therapy did not lead to any deterioration. Following IT cessation, standard outcome measures showed patients to be unremarkable, in comparison to the total HIPS patient cohort, in terms of pain severity, pain interference, psychological distress, self efficacy and employment status. Importantly, an associated reduction in health care utilization at HIPS was also found. Unfortunately there was inadequate data to compare standard outcome measures for each patient before, during and after IT therapy. This was partly due to patients being transferred from other services and also HIPS failure to collect standard outcome measures at the commencement of the study period. Widespread use of standard outcome measurement at HIPS in the latter years of the study significantly improved data availability and will help us to avoid the problem of inadequate pre and post intervention data for future case series.

Clearly it would be helpful to have evidence from randomized controlled trials to inform the use of IT therapy in chronic non-cancer pain. However, to our knowledge, no such trials have been performed. Even with regard to oral opioids, where drug delivery is much simpler, there are no randomised controlled trials of sufficient duration to provide clinically meaningful data about the development of tolerance and other adverse effects over time [31]. In the absence of guidance from randomized controlled trials, descriptive studies such as this need careful consideration. The lack of clear benefit from IT therapy, the high risk of harm and the apparent reinforcement of patient passivity, as demonstrated in this study, all raise concerns about the utility of the technique. Demonstration of the feasibility of ceasing IT therapy is also an important finding.

We conclude that it is possible to cease established IT analgesic therapy in chronic non-cancer patients without major problems and with the potential for a shift in therapeutic focus towards more active management. This study supports consideration of the cessation of established IT therapy as a practical management option.
